# Exploring the Gut Microbiota–Retina Axis: Implications for Health and Disease

**DOI:** 10.3390/microorganisms13051101

**Published:** 2025-05-10

**Authors:** Nicola Schiavone, Giulia Isoldi, Sara Calcagno, Elisabetta Rovida, Emiliano Antiga, Carolina Vieira De Almeida, Matteo Lulli

**Affiliations:** 1 Department of Experimental and Clinical Biomedical Sciences “Mario Serio”, Section of Experimental Pathology and Oncology, University of Florence, 50134 Florence, Italy; nicola.schiavone@unifi.it (N.S.); giulia.isoldi@unifi.it (G.I.); sara.calcagno@edu.unifi.it (S.C.); elisabetta.rovida@unifi.it (E.R.); 2 Department of Health Sciences, Section of Dermatology, University of Florence, 50139 Florence, Italy; emiliano.antiga@unifi.it; 3 Press Start SRL Società Benefit, 50134 Florence, Italy

**Keywords:** probiotic, prebiotic, postbiotic, non-coding RNAs, diabetic retinopathy, AMD, glaucoma, retinal artery occlusion, dietary interventions, fecal transplantation

## Abstract

The gut microbiota represents a rich and adaptive microbial network inhabiting the gastrointestinal tract, performing key functions in nutrient processing, immune response modulation, intestinal wall protection, and microbial defense. Its composition remains highly personalized and responsive to external influences, including lifestyle patterns, physical activity, body composition, and nutritional intake. The interactions of the gut microbiota with bodily systems are conventionally interpreted as broad systemic impacts on organ balance. Yet, emerging research—exemplified by the gut microbiota–brain axis—suggests the potential existence of more targeted and direct communication mechanisms. Dysbiosis, characterized by microbial ecosystem disturbance, generates multiple metabolic compounds capable of entering systemic circulation and reaching distant tissues, notably including ocular structures. This microbial imbalance has been associated with both systemic and localized conditions linked to eye disorders. Accumulating scientific evidence now supports the concept of a gut–retina axis, underscoring the significant role of microbiota disruption in generating various retinal pathologies. This review comprehensively investigates gut microbiota composition, functional dynamics, and dysbiosis-induced alterations, with specific focus on retinal interactions in age-related macular degeneration, diabetic retinopathy, glaucoma, and retinal artery occlusion. Moreover, the review explores microbiota-targeted therapeutic strategies, including precision nutritional interventions and microbial transplantation, as potential modulators of retinal disease progression.

## 1. Introduction

The gut microbiota plays a pivotal role in shaping immune system dynamics throughout life, influencing both immune resilience and susceptibility to age-related diseases. The lifelong adaptation of the immune system to microbial and environmental stressors leads to a progressive remodeling known as “immunosenescence”, which is closely linked to the chronic, low-grade inflammatory state termed “inflammaging”. While these processes contribute to increased vulnerability to age-related disorders, they are also associated with longevity, as demonstrated by centenarians who exhibit a finely tuned balance between pro- and anti-inflammatory responses [1].

Inflammaging is driven by a variety of endogenous and exogenous stimuli, including pathogens, cellular debris, and metabolic byproducts [2]. Among these, the gut microbiota plays a crucial role, acting as a “quasi-self” entity that interacts with the immune system to modulate inflammatory pathways [3]. This complex microbial ecosystem harbors an extensive gene repertoire that influences host metabolism, immune homeostasis, and overall physiological functions. Through dynamic bidirectional communication along the gut–brain axis, the gut microbiota interacts with the host to regulate the development and optimal function of multiple body systems, both digestive and extra-digestive [4]. This communication network involves intricate neural, hormonal, and immune signaling mechanisms, reinforcing the connection between gut microbiota composition and systemic health [5].

Dysbiosis, an imbalance in gut microbiota composition, has been implicated in the development of various age-related diseases, including neurodegenerative and ocular disorders [6]. The concept of the gut–retina axis has gained increasing attention since 2017, highlighting the potential role of gut microbiota disruption in the onset and progression of age-related macular degeneration (AMD) and other retinal conditions [7]. Moreover, recent research has broadened this perspective by considering interactions between the gut microbiota and other microbial communities, such as the ocular surface microbiota, in the pathophysiology of retinal degeneration and degenerative eye diseases [8]. Beyond AMD, gut microbiota dysbiosis has also been linked to diabetic retinopathy (DR), glaucoma, retinal artery occlusion (RAO), uveitis, and other ocular disorders [9].

Notably, accumulating evidence suggests that dietary modifications capable of reshaping gut microbiota composition may help prevent or even reverse AMD, underscoring the potential for microbiota-targeted interventions in ocular health [10]. This review integrates findings from various studies on the emerging gut–retina axis, exploring its implications for retinal diseases and potential therapeutic strategies.

## 2. The Gut Microbiota: Composition, Function, and Imbalance

The human gut microbiota is a highly complex ecosystem of microorganisms that inhabit the gastrointestinal tract in a symbiotic relationship with the host. It is primarily composed of six major phyla: Firmicutes, Bacteroidetes, Actinobacteria, Fusobacteria, Verrucomicrobia, and Proteobacteria, with the first two accounting for approximately 70–90% of the total microbial population [11]. Moreover, the role of the fungal microbiome (mycobiome) is increasingly recognized, with recent research also highlighting the intestinal virome, where the phageome constitutes its most abundant component [12], representing additional symbiotic communities within the gut microbiota. The gastrointestinal tract, particularly its lower sections, including the small intestine, cecum, and colon, harbors distinct microbial communities distributed along both longitudinal and transversal axes. These microbial populations are shaped by the unique physiological, chemical, nutritional, and immune conditions present in each region [13].

The human gut microbial community plays a fundamental role in maintaining physiological homeostasis, yet its composition and function are subject to modifications over time [14]. Many of its functions are well characterized, while others remain under investigation. Thus, despite significant advancements in our understanding, ongoing research continues to reveal new insights into its complexity and diverse roles.

One well-established mechanism of gut microbiota–host interaction is through microbial metabolites—small molecules (<1500 Da) that act as intermediates or end-products of bacterial metabolism. These compounds can be synthesized directly by microbes or arise from the transformation of dietary and host-derived substrates [15]. The gut microbiota plays a crucial role in digestion, vitamin synthesis, and the production of bioactive compounds such as short-chain fatty acids (SCFAs) [16]. This interaction between diet, the gut microbiota, and the host is highly complex and bidirectional: while dietary intake influences microbial composition, individual microbiota exhibits distinct metabolic responses to nutrients, ultimately shaping host metabolic functions [17]. By fermenting indigestible carbohydrates, for example, the gut microbiota generates SCFAs, which the host absorbs and utilizes as energy sources. These molecules can also regulate lipid metabolism and glucose homeostasis, influencing insulin sensitivity and energy expenditure. The production of SCFAs through dietary fiber fermentation is fundamental for maintaining gut barrier integrity and modulating immune function [18,19]. Moreover, SCFAs, particularly propionate and butyrate, play a crucial role in regulating how genes are turned on and off by affecting chemical changes in DNA-associated proteins. Nshanian et al. [20] showed that these SCFAs influence specific sites on histones helping to control how tightly genetic material is organized and how easily genes can be activated.

Additionally, Kopczyńska and Kowalczyk [21] demonstrated that SCFAs regulate gene expression in immune cells through epigenetic mechanisms, including DNA methylation and histone acetylation. These findings highlight the significant impact of SCFAs on inflammatory and metabolic pathways, offering deeper insight into their regulatory functions at the epigenetic level.

The gut microbiota also functions as a protective barrier against pathogenic microorganisms by competing for nutrients and adhesion sites on the gut lining. Additionally, it produces antimicrobial substances that inhibit the growth of harmful bacteria [19,22]. By interacting with gut-associated lymphoid tissue, the microbiota enhances immune responses against pathogens, further strengthening host defense mechanisms [16,22]. The gut microbiota influences both immune system modulation and central nervous system function by metabolizing dietary amino acids into bioactive compounds. For example, tryptophan metabolism leads to the production of indole derivatives, which impact the gut–brain axis and immune responses [23,24,25].

Notably, the immunomodulatory and neuroactive effects of the gut microbiota are closely tied to the metabolic fate of tryptophan, which is primarily degraded through three major enzymatic pathways: the serotonin, kynurenine, and aryl hydrocarbon receptor (AhR) ligand pathways [26]. The bioactive metabolites produced along these routes allow the gut microbiota to exert significant influence on both the host immune system and the central nervous system (CNS). In addition to tryptophan, the gut microbiota also modulates the metabolism of other amino acids—such as glutamine, methionine, and branched-chain amino acids—thereby contributing to broader immunological and neurological regulation [27].

More specifically, microbial activity involving tryptophan availability, the production of short-chain fatty acids (SCFAs), and the biotransformation of bile acids plays a central role in shaping host immunity [22,28]. These processes support immune cell maturation and antibody production, enhancing the host’s ability to respond to pathogens. A eubiotic gut microbiota is thus essential for maintaining immune homeostasis, while dysbiosis has been linked to chronic inflammation and a variety of immune-mediated disorders [22,23,24,25,26,27,28,29,30].

In the context of the gut–brain axis, the microbiota engages in bidirectional communication with the CNS, in part through the microbial production of neurotransmitters such as serotonin [31] and other neuroactive compounds capable of influencing mood and cognitive function [32]. Disruptions in microbial composition have been associated with increased vulnerability to psychiatric conditions such as anxiety and depression, as well as the development and progression of neurodegenerative diseases like Alzheimer’s disease [24,32].

Given this complex crosstalk between the gut and the brain—mediated by microbial metabolites and involving both neuronal and glial cell populations—the concept of a gut–retina axis emerges as a meaningful extension of this intricate network [33].

Numerous factors shape the composition, diversity, and abundance of the gut microbiota, including diet, genetics, health status and physiological alteration (e.g., pregnancy and menopause), medications, lifestyle, and age. The interaction between these factors shapes an individual’s microbiota, which can support a diverse and balanced microbial community linked to health or, conversely, lead to dysbiosis and an increased risk of disease. Among these determinants, diet is one of the most influential modulators of microbiota composition, directly shaping microbial communities and their functional outputs. In particular, the consumption of fermented foods, such as kimchi and kefir, has been shown to enhance microbial diversity and reduce inflammation, with systemic effects extending beyond the gastrointestinal tract, including potential benefits for conditions such as diabetes and rheumatoid arthritis [34]. Conversely, high-fiber diets may not induce rapid alterations in microbiota structure, possibly due to an insufficient abundance of fiber-degrading microorganisms [35]. Beyond macronutrients, micronutrient availability also influences microbiota composition. For instance, iron supplementation can induce metabolic shifts in *Lactobacilli* species, affecting gut health through the regulation of hypoxia-inducible factor-2 alpha (HIF-2α), a key regulator of intestinal homeostasis and gut barrier integrity [36]. Moreover, the gut microbiota itself plays a critical role in iron metabolism by facilitating cellular iron storage through the induction of ferritin, a protein that enhances iron absorption [37].

Host genetics and ethnicity also contribute to microbiota diversity. Notably, individuals from low-income countries tend to exhibit greater microbial diversity compared to those in industrialized nations, where the adoption of Westernized dietary patterns has been linked to microbiota depletion and an increased risk of metabolic disorders such as obesity [38]. The rising prevalence of noncommunicable chronic disorders in Western countries further highlights the impact of modern diet and lifestyle on gut microbiota composition. These disorders, characterized by chronic low-grade inflammation and gut microbiota imbalances, are shaped by both genetic and environmental factors, reinforcing the intricate relationship between host biology and the microbial ecosystem [39]. Urbanization and Westernization have been associated with microbiota depletion, leading to an increased susceptibility to obesity and infectious diseases. Moreover, exposure to environmental pollutants and cultural dietary practices further influence microbial diversity [40].

In this way, beyond diet and genetics, other environmental and lifestyle factors also play a pivotal role in shaping microbiota composition. Actually, it is widely recognized that lifestyle determinants, such as physical activity, smoking, and environmental exposures, exert direct effects on gut microbiota diversity. Regular exercise has been correlated with increased microbial diversity and an enrichment of beneficial bacterial taxa, whereas smoking is associated with reduced microbial diversity [41,42].

Aging also represents a key determinant of microbiota composition, often accompanied by microbial shifts that correlate with changes in physical and mental health, as well as increased frailty. Age-related shifts in the gut microbiota have been observed across diverse populations, characterized by a decline in beneficial butyrate-producing bacteria (such as *Faecalibacterium* and *Roseburia*), a reduction in microbial diversity, and an increased prevalence of low-abundance, potentially harmful taxa (including *Enterobacteriaceae*, *Streptococcaceae*, and *Staphylococcaceae*) [43,44]. These alterations have been linked to the rising incidence of chronic inflammatory conditions, underscoring the influence of modern lifestyles on immune regulation and disease susceptibility [39,45,46]. Importantly, microbiota composition is not solely dictated by chronological age but is instead shaped by a complex interplay of genetics, lifestyle, and environmental exposures. Consequently, an elderly individual adhering to a healthy lifestyle can maintain a diverse and resilient microbiota, mitigating age-related dysbiosis and its associated health risks.

Beyond all these factors, psychosocial stress, particularly social stress, has been associated with alterations in gut microbiota composition, including a reduction in anti-inflammatory microbial populations, thereby increasing inflammation and susceptibility to disease [47]. Furthermore, pharmacological agents, such as antidepressants, antibiotics, and addictive substances (e.g., alcohol and opioids), have been shown to modulate gut microbiota composition, influencing both gut health and brain function [48].

The imbalance in microbial composition and function, or dysbiosis, is increasingly recognized as a key factor in the pathogenesis of numerous chronic diseases. While genetic predisposition and environmental exposures contribute to disease onset, growing evidence suggests that disruptions in gut microbiota play a central role in tipping the balance toward inflammation and metabolic dysfunction. This imbalance can promote disease progression through multiple mechanisms, including the expansion of pathobionts that trigger inflammation [49], the depletion of beneficial microbes that support gut barrier integrity and immune tolerance, and alterations in microbial metabolite production, essential for modulating immune responses and maintaining gut health, and their decline fosters a pro-inflammatory environment that perpetuates a self-reinforcing cycle of dysbiosis and chronic inflammation.

This phenomenon is particularly relevant in the context of aging, where low-grade systemic inflammation, or inflammaging, accelerates gut microbial shifts, further exacerbating disease risk [50,51]. Restoring a balanced gut microbiota has emerged as a promising strategy for mitigating the detrimental effects of dysbiosis. Interventions such as dietary modifications, prebiotics [52], probiotics [53], and fecal microbiota transplantation [54] have demonstrated the potential to rebalance microbial communities and improve clinical outcomes in microbiota-associated disorders [55].

Is important to highlight that the gut microbiota is shaped by a multifaceted network of intrinsic and extrinsic factors, where genetics, diet, lifestyle, environmental exposures, and psychosocial influences dynamically interact to determine microbial diversity and functionality. Understanding these interconnections is crucial for developing targeted interventions aimed at preserving microbiota balance and mitigating disease risk across different life stages.

## 3. Informational Crosstalk Between Microbiota and Gut Host Cells

**Figure 1:**
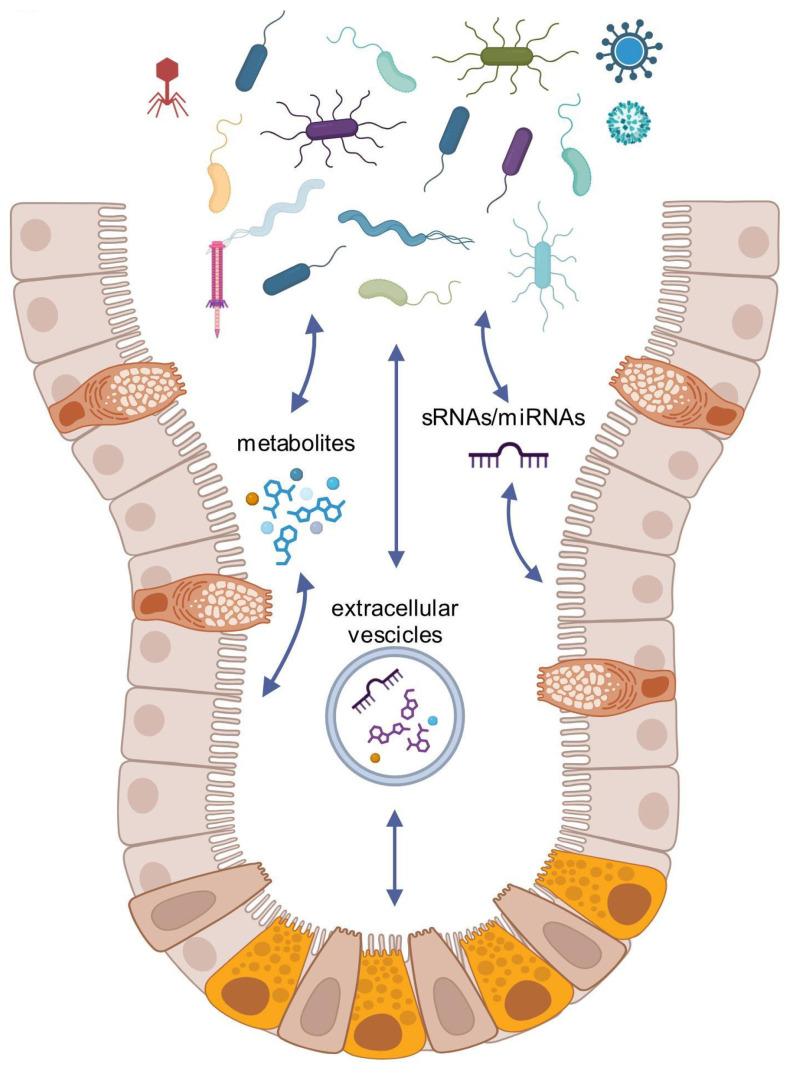
Bidirectional crosstalk between the gut and the microbiota, which occurs through intricate communication mediated by various metabolites, informational molecules, and cargo transport systems, such as extracellular vesicles (created with BioRender.com, https://app.biorender.com/, accessed on 27 March 2025).

As previously reported, the interactions between the gut and microbiota are bidirectional (Figure 1). It has been demonstrated that the microbiota may influence the composition of gut epithelial cells, both in terms of cell types and their associated functions. In particular, it affects the homeostasis of intestinal epithelial cells (IECs), as well as their ability to absorb nutrients, produce mucus and antimicrobial peptides, and secrete immune mediators and hormones. In turn, IECs shape the microbiota, ensuring its uniqueness and balance through the production of various bioactive molecules [56].

Beyond metabolites and other signaling molecules, the composition of the microbiota is also specifically regulated in two ways: horizontally, through small RNAs (sRNAs), which is not further discussed in this review, and vertically, via crosstalk between colon cells—primarily enterocytes—and bacteria, mediated by microRNAs (miRNAs) and possibly other sRNAs [57,58] (Figure 1).

### 3.1. Gut-to-Microbiota Talk

The bidirectional communication between gut epithelial cells and microbiota involves a sophisticated molecular dialogue mediated by miRNAs. As depicted in Figure 1, eukaryotic cells produce specific miRNAs in response to bacterial metabolites, which can regulate bacterial gene expression and growth, a mechanism fundamental to establishing microbiota uniqueness. This intricate intercellular communication has emerged as a critical pathway in understanding microbial dynamics and disease progression; however, the only evidence of a direct effect on bacterial growth and growth repression was provided by Liu et al. [59]. Pioneering research has revealed that miRNA-based bacterial regulation plays a significant role in pathological conditions, particularly in cancer and inflammatory intestinal diseases. Although the internalization of miRNAs by bacteria appears non-specific, their action is remarkably precise, targeting specific genes and creating distinctive microbiota-based disease signatures [60,61,62,63,64,65]. For instance, a groundbreaking study in gastric cancer proposed using these molecular signatures as a potential non-invasive diagnostic screening tool [66].

Interestingly, miRNA patterns have been demonstrated to reflect both dietary intake and microbiota composition, offering a promising avenue for monitoring dietary impacts on microbial ecosystems [67]. Dietary functional oligosaccharides, such as stachyose found in plants, play a crucial role in modulating this miRNA-mediated communication. When reaching the colon undigested, stachyose interacts with intestinal epithelial cell membrane receptors, subsequently downregulating specific miRNAs produced in nanovesicles. This process can suppress certain bacterial species, like *Lactobacilli,* effectively shaping the microbiota composition [68]. These miRNAs function as sophisticated microbiota modulators, maintaining their potential to influence microbial communities even when supplemented in food. Ultimately, functional oligosaccharides like stachyose are metabolized in the colon, simultaneously acting as prebiotics and molecular regulators of microbial ecosystems [68]. Moreover, evidence suggests that plant-derived microRNAs (miRNAs) can directly influence the composition of the gut microbiota and strengthen intestinal barrier integrity [69].

### 3.2. Microbiota-to-Gut Talk

It is well established that the microbiota plays a crucial role in modulating gastrointestinal tract cells, significantly enhancing barrier function through multiple mechanisms [58,70]. These include stimulating mucus production, upregulating tight junction proteins (such as ZO-1 and E-cadherin), and maintaining the stem-cell niche [71]. Bacteria within the microbiota achieve these functions by leveraging various metabolites and signaling molecules, including SCFAs and host-induced miRNAs (Figure 1). While the unidirectional communication from host to microbiota has been recently partially elucidated, the understanding of informational signal exchange from microbes to host remains limited. The current literature predominantly focuses on pathogenic microbial effects [72,73].

However, emerging evidence suggests more complex interspecies communication mechanisms. A notable example is the symbiotic relationship between the squid *Euprymna scolopes* and the bacterial symbiont *Vibrio fischeri*, where specific small RNA (sRNA) transfer has been observed to enhance host fitness [74]. The presence of sRNA in human circulation is now a well-documented phenomenon [75], which will be further discussed subsequently. These observations prompt a compelling hypothesis: the informational flux from symbiotic bacteria to gut or other body cells may represent an underexplored and potentially significant biological mechanism.

## 4. The Gut Microbiota–Retina Axis: Merging Concept and Mechanisms

**Figure 2:**
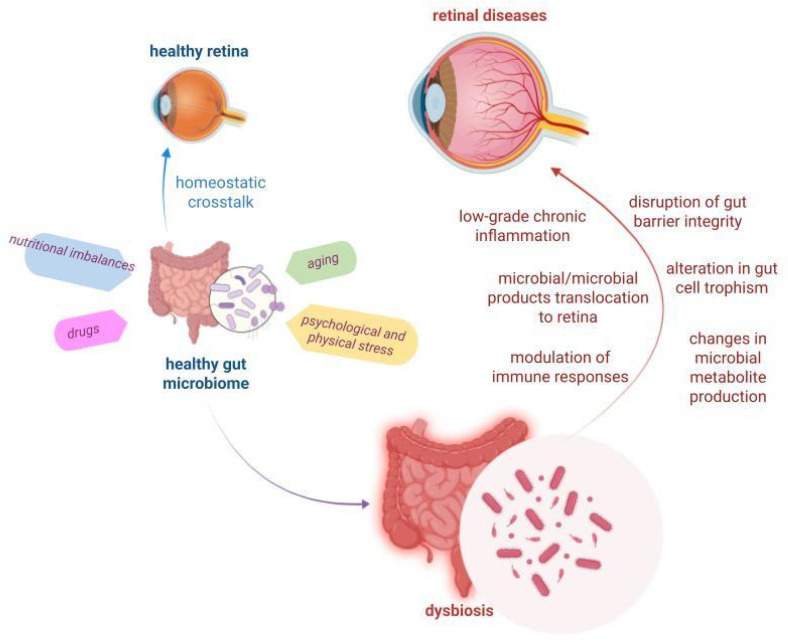
The gut microbiota–retina axis. The gut microbiota plays a regulatory role in retinal homeostasis. Numerous stressors—such as nutritional imbalances, drugs, aging, psychological and physical stress—can lead to dysregulation in both the composition and function of the gut microbiota. The resulting dysbiosis can disrupt crosstalk with the retina, influencing the pathogenesis of various retinal diseases through multiple mechanisms, including alteration in gut cell trophism, changes in microbial metabolite production, modulation of immune responses, the establishment of low-grade chronic inflammation, disruption of gut barrier integrity, and translocation of microbes/microbial products to the retina (created with BioRender.com, https://app.biorender.com/, accessed on 27 March 2025).

Beyond well-characterized host–microbiota interactions and associated pathologies, contemporary research has increasingly focused on the intricate interplay between gut microbiota and retinal health. The emerging concept of the gut–retina axis postulates that the gut microbiota exerts a regulatory influence on retinal homeostasis, underscoring its potential significance in ocular physiology and disease progression [7,16]. As depicted in Figure 2, this homeostatic crosstalk could be affected by numerous stressors, such as nutritional imbalances, drug assumption, aging, and psychological and physical stress, which cooperate in the onset of dysbiosis. This paradigm has prompted intensive investigation into the underlying mechanisms, particularly in the context of potential therapeutic interventions and nutritional strategies [76,77].

The primary mechanisms of interaction involve multifaceted modulation of systemic processes, including (i) inflammatory response regulation, (ii) immune system modulation, (iii) gut–blood barrier integrity maintenance, and (iv) cellular trophism (Figure 2). These processes are mediated through complex interactions involving endotoxins, metabolites, reactive oxygen species (ROS), and cytokine induction. While traditionally considered indirect, emerging evidence suggests the possibility of direct molecular and informational exchange between microbiota and retinal cells, analogous to the established gut–brain axis mechanisms [78,79,80]. The potential complexity of these interactions may remain partially unexplored due to existing technical limitations and the absence of sophisticated models capable of investigating intricate interkingdom molecular networks connecting distant anatomical compartments in multicellular organisms.

Both Gram-positive and Gram-negative bacteria produce extracellular vesicles (EVs), with outer membrane vesicles (OMVs), with Gram-negative bacteria being more extensively characterized. These EVs serve as sophisticated molecular transporters, capable of carrying diverse biomolecules, including toxins, proteins, neurotransmitters, lipids, and genetic material such as chromosomal DNA and small RNAs (sRNAs) with gene-regulatory properties analogous to miRNAs [78,81]. EVs demonstrate remarkable intercellular communication capabilities, participating in gut–brain axis signaling through multiple pathways. They can interact directly with neuronal or mucosal cells locally or enter systemic circulation, potentially crossing biological barriers to deliver biomolecules to distant organs. Experimental evidence in mice has demonstrated OMVs administered orally can be detected not only in gut epithelial cells but also in distant organs, including the heart, liver, kidney, spleen, and brain, and have been identified in blood samples from healthy human donors [82,83]. Moreover, these vesicles play critical roles in immune and inflammatory modulation [84,85].

sRNAs represent another fascinating molecular mechanism, capable of indirectly regulating pathogenicity-related gene expression by interacting with host cellular proteins [86]. Emerging research suggests bacterial sRNAs transported via EVs can directly modulate gene expression in eukaryotic cells. Preliminary studies have demonstrated bacterial transfer RNA (tRNA) fragments (tRFs) can be transferred to eukaryotic cells in vitro [87,88], suggesting potential direct molecular communication mechanisms previously unanticipated. These collective findings propose that, similar to brain interactions, the neuroretina may be susceptible to influence by EV-transported molecular cargo, warranting extensive further investigation.

Additional investigations have revealed compelling evidence of bacterial translocation and presence in various systemic contexts. Some studies indicate that commensal bacteria can enter systemic circulation, with DNA fingerprinting identifying their presence in atheromatous plaques [89,90]. While the pathological implications for ocular diseases remain largely unexplored, these findings present intriguing avenues for future research. More recent developments have documented bacterial presence in retinal lesions associated with degenerative diseases, including retinitis pigmentosa and congenital amaurosis. Notably, these microbial presences correlate with specific genetic mutations, particularly in the crumbs homolog 1 (*CRB1*) gene. Such mutations compromise the integrity of both the blood–retinal barrier (BRB) and intestinal epithelial barrier, potentially facilitating bacterial colonization [91].

Beyond the bacteriome, comprehensive microbiota research necessitates consideration of the virome and mycobiome. The Gut Phage Database (Sanger Institute) reveals the human intestine harbors over 140,000 distinct phages, approximately half previously uncharacterized. While direct associations with ocular diseases remain unestablished, phage dysbiosis has been implicated in metabolic and inflammatory conditions known to impact ocular health [92,93,94,95]. The mycobiome represents another critical component despite constituting a relatively minor proportion of gut microbiota. Fungi play a pivotal role in maintaining microbiota equilibrium [96,97,98,99]. Groundbreaking research by Jayasudha et al. [100] and Padakandla et al. [101] demonstrated direct correlations between mycobiome alterations and diabetic retinopathy in both human and animal models.

## 5. Retinal Diseases and Gut Microbiota

### 5.1. Age-Related Macular Degeneration

Age-related macular degeneration (AMD) is a complex eye disorder that affects one in eight individuals aged 60 or older and is the leading cause of irreversible blindness in the elderly worldwide. AMD is characterized by the progressive neurodegeneration of photoreceptor and retinal pigment epithelial (RPE) cells at the macula, the central region of the retina. The disease is associated with inflammation, oxidative stress, and vascular dysfunction. AMD is classified into two forms: dry (non-exudative) and wet (exudative). Dry AMD involves drusen deposition, RPE hyperpigmentation, and atrophy, while wet AMD results from the abnormal growth of blood vessels beneath the retina. Despite affecting only 10% of AMD patients, wet AMD is responsible for approximately 90% of AMD-related vision loss [102].

Growing evidence highlights the role of gut microbiota dysbiosis in AMD pathogenesis, particularly through low-grade chronic inflammation driven by impaired intestinal barrier function [103,104]. Mendelian randomization studies have established a causal relationship, identifying specific microbial taxa associated with dry AMD [10]. Notably, the family *Peptococcaceae* and the genera *Bilophila*, *Faecalibacterium*, and *Roseburia* were linked to an elevated AMD risk, whereas *Candidatus Solaeferrea*, genus *Desulfovibrio*, and genus *Eubacterium ventriosum group* [105] emerged as protective factors. Comparative β-diversity analyses further revealed distinct gut microbial profiles in wet AMD patients, who exhibited significantly reduced alpha diversity. Intriguingly, conflicting findings have been reported regarding Bacteroidota levels, while Zhang et al. [103] observed an increase in wet AMD and Baldi et al. [104] documented reduced proportions of Bacteroidota, Bacteroidales, and *Prevotellaceae*. This highlights that, while dysbiosis is a characteristic feature in AMD patients, a definitive microbial signature remains elusive. Additionally, it cannot be excluded that dysbiosis is one of several factors to consider and that alterations in specific microbial profiles should be contextualized alongside other factors when determining their impact on retinal pathophysiology. Nevertheless, different methodological approaches could yield somewhat divergent results, depending, for example, on differences in the sampled population and sample variability

Building upon the systemic relevance of SCFAs, emerging evidence suggests their influence extends beyond the gut; in particular, they demonstrated ocular bioavailability. Experimental studies in mice confirmed that intraperitoneally administered SCFAs can reach ocular tissues, where they attenuate intraocular inflammation, indicating their capacity to cross the blood–eye barrier [106]. Interestingly, patients with age-related macular degeneration (AMD) exhibit a marked reduction in its production, with wet AMD cases showing decreased abundance of SCFA-producing microbes such as *Phascolarctobacterium* and *Parabacteroides* [107,108]. These findings suggest that the anti-inflammatory properties of SCFAs may have therapeutic relevance not only in intestinal and systemic contexts, as outlined in the previous section, but also in ocular health.

### 5.2. Diabetic Retinopathy

Diabetic retinopathy (DR), a leading cause of blindness in diabetes, arises from chronic hyperglycemia-induced metabolic dysregulation, oxidative stress, and inflammation, ultimately triggering retinal vascular damage, microangiopathy, and neovascularization [109]. While metabolic and vascular mechanisms dominate DR research, emerging evidence implicates gut microbiota dysbiosis in disease progression. Systemic inflammation, a hallmark of diabetes, is exacerbated by gut dysbiosis, which promotes immune dysregulation and accelerates retinal vascular injury [110]. DR patients exhibit altered gut microbial profiles, including reduced Actinobacteria and Bacteroidetes alongside elevated *Escherichia*, *Faecalibacterium*, and *Prevotella* [111,112]. Contrasting reports note increased Bacteroidetes in some DR cohorts [112,113], underscoring the lack of a consistent microbial signature, as previously mentioned regarding AMD. Mendelian randomization analyses identified *Christensenellaceae* and *Peptococcaceae* as protective taxa, whereas *Eubacterium rectale*, *Adlercreutzia*, and *Ruminococcaceae* UCG-011 were associated with higher DR risk [114], suggesting microbiota-mediated modulation of disease susceptibility. There are clinical studies aimed at altering the composition and/or function of the gut microbiota in individuals with retinal diseases, through the administration of, for example, prebiotics, probiotics, and postbiotics, with significant clinical improvements reported. These results will be discussed in more detail in the subsequent chapter on the therapeutic potential of the microbiota.

It is important to highlight that, in a dysbiotic state, metabolic homeostasis is also disrupted, with a decrease in key metabolites such as SCFAs, potentially compromising gut barrier integrity and amplifying inflammatory cascades [115]. As demonstrated by Qin et al. [116], patients with diabetic retinopathy (DR) exhibit specific microbial signatures, including reduced abundance of *Butyricicoccus* and *Ruminococcus torques*, along with lower plasma SCFA levels. Machine learning models using these microbial and genomic features have shown predictive value for DR onset, linking dietary patterns and peripheral immune profiles to dysbiosis-driven pathogenesis.

### 5.3. Glaucoma

Glaucoma, a leading cause of irreversible blindness, encompasses a group of optic neuropathies defined by progressive retinal ganglion cell (RGC) loss and optic nerve degeneration [117]. Mechanisms driving RGC damage include oxidative stress, mitochondrial dysfunction, excitotoxicity, and neuroinflammation. Although elevated intraocular pressure (IOP) is a major risk factor, normal-tension glaucoma, characterized by optic neuropathy despite normal IOP, highlights the disease’s multifactorial nature. Beyond genetic, vascular, and IOP-related mechanisms, emerging research implicates gut microbiota dysbiosis in glaucoma pathogenesis.

Comparative analyses reveal distinct gut microbial profiles in glaucoma patients, marked by reduced α-diversity and altered β-diversity. Gong et al. [118] reported elevated *Prevotellaceae*, *Escherichia coli*, and unclassified *Enterobacteriaceae*, alongside depleted *Megamonas* and *Bacteroides plebeius*, in glaucoma cohorts. These dysbiotic shifts correlated with perturbations in serum metabolites, including amino acids, hormone derivatives, and bile acids. Mendelian randomization studies further identified *Eubacterium* (nodatum group), *Lachnospiraceae* (NC2004 group), *Roseburia*, and *Ruminococcaceae* (UCG004) as potential microbial modulators of glaucoma risk [119]. However, Li et al. [120] found no definitive causal relationship, underscoring the complexity of microbiota–glaucoma interactions.

Immune-mediated mechanisms further link gut microbiota to glaucomatous neurodegeneration. Chen et al. [121] demonstrated that gut microbiota-sensitized T cells play a pivotal role in RGC degeneration. In a murine glaucoma model, transient IOP elevation induced retinal T-cell infiltration and subsequent neurodegeneration that persisted beyond IOP normalization. Crucially, molecular mimicry between microbial and human proteins, in particular with heat shock proteins (HSPs), was identified as the triggering mechanism. In this scenario, HSPs from gut microflora have been identified as the target antigen of memory T cells, which, after reaching the retina, become activated by retinal HSPs upregulated during IOP elevation. Germ-free mice, lacking commensal microbiota, were protected from both T-cell responses and neurodegeneration, underscoring the gut–retina axis in glaucoma pathogenesis.

### 5.4. Retinal Artery Occlusion

Retinal artery occlusion (RAO), an ophthalmologic emergency analogous to stroke, results from partial or complete obstruction of the central retinal artery, leading to irreversible retinal ischemia and vision loss [122]. While traditional risk factors (hypertension, diabetes, atherosclerosis) dominate RAO pathogenesis, emerging evidence implicates gut microbiota dysbiosis in disease development.

The thromboembolic nature of RAO suggests shared mechanisms with atherosclerosis, where gut-derived microbial translocation may exacerbate endothelial dysfunction and systemic inflammation. Zysset-Burri et al. [123] identified RAO-specific dysbiosis, characterized by reduced Bacteroidetes and elevated Proteobacteria, with particular enrichment of Actinobacteria, *Bifidobacterium* spp., *Bacteroides stercoris*, and *Faecalibacterium prausnitzii*. Conversely, *Odoribacter*, *Parasutterella*, and *Lachnospiraceae* were significantly depleted. Mendelian randomization analyses corroborated these findings, identifying two microbial taxa as risk factors and five as protective, including anti-inflammatory *Bacteroides* and *Burkholderia* [124,125].

Notably, microbial DNA detected within atherosclerotic plaques [126] supports the “gut–retina–vascular axis” hypothesis, where bacterial translocation promotes arterial inflammation [127]. Actinobacteria components are disproportionately represented in plaques [89], suggesting their direct involvement in RAO-associated atherosclerosis. Furthermore, elevated levels of the gut-derived proatherogenic metabolite trimethylamine-N-oxide (TMAO) in RAO patients [123] mechanistically link microbial metabolism to vascular pathology.

## 6. Therapeutic Potential of Microbiota: Targeted Interventions in Retinal Diseases and Future Directions

Growing evidence of the gut–retina axis in ocular disease pathogenesis has spurred interest in microbiota-targeted therapies, including probiotics, prebiotics, postbiotics, dietary interventions, and fecal microbiota transplantation (FMT). Personalized approaches may optimize outcomes given dysbiosis heterogeneity [16,128].

### 6.1. Probiotics

Probiotics, operationally defined as viable microorganisms that confer measurable health benefits when administered in sufficient quantities [129], have emerged as promising therapeutic candidates for retinal diseases through their capacity to modulate gut microbiota composition and function. The most clinically relevant probiotic strains encompass select species within the *Lactobacilli* and *Bifidobacterium* genera, complemented by specific bacterial strains from *Streptococcus*, *Enterococcus*, and *Bacillus* genera, as well as certain *Saccharomyces* yeast species [130]. While some of these microorganisms are already under investigation for their therapeutic potential in retinal disorders, others remain to be explored, offering opportunities to expand the probiotic landscape in this context.

Crucially, the effects of probiotics are highly strain-specific, and their therapeutic properties cannot be generalized across entire genera or species [131]. This specificity underscores the importance of targeted selection and characterization of probiotic strains for clinical application.

Mechanistically, probiotics mediate their beneficial effects through multiple pathways: (1) reinforcement of intestinal epithelial barrier function via tight junction protein upregulation, (2) immunomodulation through cytokine network regulation, (3) enhancement of cellular stress resistance mechanisms, and (4) competitive exclusion of pathogenic microorganisms [11,132,133,134]. These processes, initiated in the gut, exert systemic consequences that extend to distal organs such as the retina. In particular, systemic reductions in chronic inflammation and oxidative stress—two key pathological hallmarks of retinal degenerative diseases—may represent critical mediators linking gut probiotic activity to retinal protection. Thus, probiotics may influence the gut–retina axis by fostering a microbiome environment that promotes anti-inflammatory and neuroprotective states.

Preclinical investigations have provided compelling evidence of this concept [135]. Verma et al. [136] demonstrated that recombinant *Lactobacillus paracasei* engineered to express human angiotensin-converting enzyme 2 (ACE2) significantly attenuated key pathological features in diabetic retinopathy models, including reduction in retinal proinflammatory cytokine expression, decreased acellular capillary formation, and preservation of retinal ganglion cell (RGC) density. Complementary work by Morita et al. [53] showed that administration of *Lactobacillus paracasei* KW3110 effectively suppressed retinal inflammation by modulating macrophage populations—specifically, by reducing proinflammatory cytokine-producing macrophages and preventing age-related retinal cell loss. Building on these findings, subsequent research using heat-killed *Lactobacillus paracasei* KW3110 demonstrated its ability to promote anti-inflammatory M2 macrophage polarization in human subjects, leading to increased production of interleukin-10 (IL-10) [137].

Notably, both M2 macrophage-conditioned media and purified IL-10 exhibited significant neuroprotective effects against light-induced photoreceptor apoptosis in experimental models. Lastly, modulation of the gut microbiota in type 1 diabetic mice through a four-month administration of *Lactobacillus rhamnosus* resulted in significant ocular health benefits, including reduced intraocular pressure and improved blood glucose levels compared to the control group [138].

While these findings underscore the therapeutic promise of probiotics for retinal diseases, several challenges must be addressed to facilitate clinical translation. These include the identification of optimal strains and determination of effective dosing regimens. Robust human trials are essential to validate efficacy and elucidate the underlying mechanisms [53]. Additionally, given the cooperative and dynamic nature of the gut microbiota, future approaches may benefit from employing multi-strain probiotic formulations designed to reshape the microbial ecosystem in a synergistic and sustained manner [139].

### 6.2. Prebiotics

Prebiotics represent a class of nondigestible dietary substrates that undergo selective microbial fermentation in the gastrointestinal tract, thereby conferring measurable physiological benefits to the host through targeted modulation of gut microbiota composition and function [140].

Although the therapeutic use of prebiotics in ophthalmology is still at an early stage, certain compounds have demonstrated promising retinal-protective effects through gut-mediated mechanisms by promoting the proliferation of beneficial, anti-inflammatory bacterial taxa [141]. Among these, chitosan oligosaccharides (COS) have exhibited multimodal retinal protective properties, including (1) enhancement of functional recovery following oxidative insult, (2) preservation of retinal laminar architecture, and (3) dose-dependent neuroprotection of retinal neurons. These effects appear mechanistically linked to COS-mediated attenuation of reactive oxygen species, upregulation of endogenous antioxidant defenses, and suppression of nuclear factor kappa-B (NF-κB) signaling pathways [142]. Notably, in experimental autoimmune uveoretinitis models, COS administration significantly reduced inflammatory cell infiltration into vitreoretinal tissues through NF-κB-dependent downregulation of proinflammatory mediators, further supporting its anti-inflammatory potential via gut–immune–retina signaling pathways [143]. Furthermore, COS has demonstrated cytoprotective effects against blue light-induced phototoxicity in RPE cells, suggesting potential applications in age-related macular degeneration [144].

The polyphenolic compound resveratrol, while not traditionally classified as a prebiotic, exhibits complementary microbiota-modulating properties coupled with direct retinal protective effects. Its pleiotropic mechanisms of action encompass: (1) oxidative stress mitigation through free radical scavenging, (2) suppression of proinflammatory cascades, (3) maintenance of mitochondrial bioenergetics, (4) inhibition of apoptotic pathways, and (5) modulation of angiogenic signaling—all of which constitute critical pathophysiological pathways in various retinopathies [145,146]. Moreover, evidence from human studies adds further weight to the prebiotic potential of certain dietary components. A pioneering clinical trial by Tzounis et al. [147] demonstrated that flavanol-rich cocoa consumption significantly enhanced the growth of beneficial gut microbes, particularly *Lactobacilli* and *Bifidobacterium* spp., thereby reinforcing the concept that dietary polyphenols can act as functional prebiotics with systemic and possibly retinal benefits.

In conclusion, prebiotics represent a promising, though still underexplored, therapeutic avenue for retinal diseases through their capacity to reshape the gut microbiome and modulate systemic inflammatory and neuroprotective pathways. Future research should focus on identifying specific prebiotic compounds with retinal efficacy, optimizing dosage and administration protocols, and validating their benefits in clinical settings.

### 6.3. Postbiotics

Postbiotics represent a novel class of bioactive compounds derived from inactivated microorganisms or their structural components, which have been shown to confer significant physiological benefits to the host organism through microbiota-related mechanisms [148]. These preparations encompass a diverse array of microbial products, including cell-free supernatants containing secreted metabolites, complex exopolysaccharides, functional enzymes, microbial cell wall fragments, SCFAs, bacterial lysates, and other metabolic byproducts of gut microbiota activity [149].

Through the gut–retina axis, postbiotics may influence ocular health by (1) direct antimicrobial effects against pathogenic organisms, (2) potent antioxidant properties, (3) immunomodulatory capacity to regulate both innate and adaptive immune responses, and (4) enhancement of intestinal barrier integrity through structural and functional modifications [150]. Similar to SCFAs, secondary bile acids such as ursodeoxycholic acid (UDCA) and tauroursodeoxycholic acid (TUDCA) have demonstrated protective effects in models of DR. Notably, UDCA has been shown to suppress retinal inflammation, restore tight junction proteins (e.g., claudin-1 and claudin-19), and preserve BRB function in streptozotocin (STZ)-induced diabetic mice [151]. UDCA also attenuates endoplasmic reticulum stress and enhances pericyte survival—critical elements in maintaining retinal vascular stability [152]. Tryptophan-derived metabolites represent another important class of postbiotic compounds within the gut–retina axis. Kynurenic acid, a terminal product of the kynurenine, possesses anti-inflammatory and neuroprotective properties. Its ability to cross both the blood–brain and BRB makes it a compelling candidate for treatment of retinal aging and neurodegeneration [153,154].

Compared to traditional probiotic formulations, postbiotic preparations offer several distinct therapeutic advantages for ophthalmic applications, such as (1) enhanced bioavailability due to their pre-processed nature, (2) more consistent and reproducible pharmacological effects, and (3) elimination of risks associated with horizontal gene transfer of antibiotic resistance determinants [150,154]. However, while these properties make postbiotics an attractive therapeutic modality, significant research gaps remain regarding their specific applications in retinal pathology. Comprehensive preclinical and clinical studies are required to fully elucidate their efficacy profiles, optimal dosing regimens, and long-term safety parameters in ophthalmic conditions [154].

### 6.4. Dietary Interventions

The nutritional composition of dietary intake serves as a fundamental determinant of gut microbiota ecology, with profound implications for systemic health and retinal homeostasis. As previously established, dietary patterns enriched with probiotic and prebiotic components foster the proliferation of beneficial microbial taxa, thereby enhancing the endogenous production of SCFAs [155].

The Mediterranean dietary pattern, characterized by high consumption of vitamins C and E, zinc, carotenoids (beta-carotene, lutein, zeaxanthin), vitamin D, and omega-3 polyunsaturated fatty acids (PUFAs), has demonstrated particular efficacy in promoting retinal health [156]. Clinical evidence indicates this dietary regimen may significantly attenuate the progression of AMD, especially when combined with targeted antioxidant supplementation [157,158,159]. The landmark AREDS study by Chew et al. [160] documented a 25% reduction in AMD progression risk following five years of combined vitamin C/E, beta-carotene, and zinc supplementation. Complementary preclinical research by Prokopiou et al. [161] revealed that omega-3 PUFA administration in aged murine models reduced pathological lipofuscin accumulation in the retinal pigment epithelium while protecting against age-related photoreceptor degeneration. The omega-3 derivatives eicosapentaenoic acid (EPA) and docosahexaenoic acid (DHA) mediate these protective effects through multiple pathways: (1) enhancement of cellular oxidative stress resistance, (2) suppression of inflammatory cascades, and (3) modulation of critical cell survival signaling networks [162]. Furthermore, clinical investigations by Díaz-López et al. [163] demonstrated that Mediterranean diet supplementation with extra virgin olive oil significantly reduced diabetic retinopathy incidence compared to low-fat controls, attributable to its high concentration of bioactive phenolic compounds with antioxidant and anti-inflammatory properties.

Intermittent fasting (IF) regimens have emerged as another promising dietary intervention for retinal protection. Beli et al. [164] demonstrated that IF induces significant gut microbiota remodeling in db/db diabetic mice, characterized by increased Firmicutes/Bacteroidetes ratios and consequent elevation of neuroprotective secondary bile acids like TUDCA. These microbial changes correlated with reduced retinal microglial activation, decreased leukocyte infiltration, and attenuated formation of acellular capillaries. Epidemiological findings by Choi et al. [165] further associate IF with reduced AMD risk, particularly in individuals <70 years and those with obesity. Preclinical studies suggest IF may also protect against glaucoma through reduction in retinal ganglion cell apoptosis [166], mediated through multiple mechanisms: (1) improved insulin sensitivity, (2) reduced circulating glucose and lipids, (3) suppression of proinflammatory mediators, and (4) enhanced autophagic flux—collectively preserving retinal microvascular integrity and Müller cell function in diabetic models [167].

Conversely, the Western dietary pattern—characterized by excessive consumption of red meat, saturated fats, processed foods, and sugar-sweetened beverages—promotes gut dysbiosis, intestinal barrier dysfunction, and systemic inflammation that accelerate retinal pathology [168,169]. Keeling et al. [170] demonstrated that chronic Western diet feeding induces early-intermediate AMD-like phenotypes in murine models, including drusen-like deposits and retinal pigment epithelium abnormalities, highlighting the critical role of nutritional factors in retinal disease prevention.

The multiple studies outlined above—spanning the Mediterranean diet, intermittent fasting, and Western diet patterns—highlight how targeted nutritional strategies can influence gut-derived bioactive metabolites and, through the gut–retina axis, modulate retinal inflammation, vascular integrity, and neurodegeneration. These mechanisms support the hypothesis that diet is not merely a modifiable risk factor but may function as a foundational element in preventive and adjunctive therapeutic strategies for retinal diseases.

### 6.5. Fecal Microbiota Transplantation

Fecal microbiota transplantation (FMT) represents an innovative therapeutic approach involving the transfer of minimally processed fecal microbiota from rigorously screened healthy donors to recipients, with the objective of restoring normative gut microbial diversity and metabolic function [171,172]. Originally developed as a highly effective treatment for recurrent *Clostridioides difficile* infection, this procedure has demonstrated therapeutic potential for numerous extra-intestinal conditions, including ocular pathologies [173].

Recent preclinical studies support the hypothesis that restoring gut microbial homeostasis via FMT can exert protective effects on the retina. For instance, Parker et al. [174] demonstrated that transplantation of aged donor microbiota into young recipients precipitated accelerated retinal and central nervous system inflammation, characterized by elevated proinflammatory cytokine signaling and functional impairment of retinal proteins. These pathological changes were mechanistically linked to increased intestinal barrier permeability. Conversely, the introduction of microbiota from young donors into aged recipients mitigated retinal degeneration and inflammation.

These findings underscore the therapeutic potential of FMT in targeting systemic and ocular disease pathways, offering a promising strategy for retinal conditions such as age-related macular degeneration and other inflammation-driven retinopathies. By reestablishing gut microbial homeostasis, FMT may support retinal integrity and reinforce the concept of the gut–retina axis as a viable target in ophthalmic disease prevention and therapy.

From a safety perspective, while FMT is generally well tolerated, significant risks warrant careful consideration [175]. Potential adverse effects include (i) acute complications related to microbial translocation; (ii) long-term ecological consequences of microbiota alteration; and (iii) risk of sepsis in immunocompromised populations. These safety concerns underscore the necessity for rigorous donor screening protocols and careful patient selection, particularly when considering FMT for vulnerable populations with retinal disorders.

Current evidence positions FMT as a promising investigational therapy for retinal diseases, though further research is required to (i) standardize preparation and administration protocols; (ii) establish optimal dosing regimens; (iii) validate long-term efficacy and safety profiles; and (iv) identify predictive biomarkers for treatment response. The mechanistic insights gained from these early studies highlight the profound influence of gut microbial ecology on retinal homeostasis and provide a strong rationale for continued investigation of FMT in ophthalmic therapeutics.

## 7. Conclusions

The growing recognition of the gut–retina axis represents a fundamental advancement in our understanding of ocular pathophysiology, establishing the intestinal microbiota as a critical modulator of retinal homeostasis and disease. This complex bidirectional communication network, mediated through immunological, metabolic, and neuroendocrine pathways, underscores how gut dysbiosis can propagate systemic low-grade inflammation and metabolic derangements that ultimately compromise retinal function. The mechanistic elucidation of these processes has unveiled novel therapeutic opportunities, positioning microbiota-targeted interventions—including probiotics, prebiotics, postbiotics, dietary modifications, and fecal microbiota transplantation—as promising strategies to restore microbial–immune–metabolic equilibrium and mitigate retinal pathology.

However, while preclinical and early clinical evidence is compelling, the translational potential of these approaches necessitates rigorous validation through controlled trials to establish standardized protocols, safety profiles, and predictive biomarkers. The gut–retina paradigm not only expands our etiological understanding of ocular diseases but also challenges traditional therapeutic boundaries by integrating systemic and local mechanisms of disease modulation.

It is crucial to recognize the limitations of the current findings in gut microbiota research, as the field presents contrasting and often contradictory results. For example, while high microbial diversity is typically associated with health, there are cases where increased diversity does not correlate with more favorable phenotypic outcomes. This is especially evident in polyphenol intervention studies, where inconsistent results hinder the formulation of definitive conclusions. Furthermore, many claims regarding the relationship between gut microbiota and improvements in retinal diseases rely on phylum- and genus-level classifications, which are inherently broad and may overlook the subtleties of strain-level differences. Additionally, the classification of viral genomes presents another challenge, as the identification of ‘beneficial’ versus ‘deleterious’ viruses remains ambiguous. These complexities highlight the need for a more refined approach to microbiota research, one that emphasizes strain-level resolution and more comprehensive assessments of microbial and viral interactions

As this field evolves, a deeper interrogation of microbial–host crosstalk will be essential to refine these interventions and unlock their full clinical potential, ultimately paving the way for more holistic and precision-based management of retinal disorders. This conceptual shift underscores the imperative for interdisciplinary collaboration between ophthalmology, microbiology, and immunology to harness the therapeutic promise of the gut–retina axis while addressing its inherent biological complexities.

## Data Availability

No new data were created or analyzed in this study.
